# Correction: Beyond cysts – organization of epithelial networks in the murine thymus

**DOI:** 10.1242/jcs.264607

**Published:** 2025-12-18

**Authors:** Stepan Vodopyanov, Leslie Gunther-Cummins, Sophia DesMarais, Maria K. Lagou, Xheni Nishku, Joseph Churaman, Hillary Guzik, Rotem Alon, Vera DesMarais, Frank Macaluso, George S. Karagiannis

**Affiliations:** ^1^ Department of Microbiology and Immunology, Albert Einstein College of Medicine, Bronx, NY 10461, USA; ^2^ Integrated Imaging Program for Cancer Research, Montefiore-Einstein Comprehensive Cancer Center, Bronx, NY 10461, USA; ^3^ Analytical Imaging Facility, Albert Einstein College of Medicine, Bronx, NY 10461, USA; ^4^ Department of Cell Biology, Albert Einstein College of Medicine, Bronx, NY 10461, USA; ^5^ Gruss-Lipper Biophotonics Center, Albert Einstein College of Medicine, Bronx, NY 10461, USA; ^6^ Cancer Dormancy Institute, Montefiore-Einstein Comprehensive Cancer, Center, Bronx, NY 10461, USA; ^7^ Marilyn and Stanley M. Katz Institute for Immunotherapy for Cancer and Inflammatory Disorders, Montefiore-Einstein Comprehensive Cancer Center, Bronx, NY 10461, USA; ^8^ Tumor Microenvironment and Metastasis Program, Montefiore-Einstein Comprehensive Cancer Center, Bronx, NY 10461, USA

There was an error in *J. Cell Sci.* (2025) **138**, jcs264079 (doi:10.1242/jcs.264079).

Following the departure of Maria K. Lagou from the laboratory, her contributions were mis-characterized. Upon reevaluation, it was shown that she worked on several animals and generated tissues that were subsequently used for the characterization of the reported respiratory and epithelial networks within the murine thymus by other authors of the manuscript.

Hence, the author list has been adjusted to add her as an author as given above and as updated in the original paper.

The Acknowledgements has been updated to remove the sentence ‘We would also like to acknowledge Dr Maria K. Lagou, for technical support’ and the Author Contributions section has been updated to add M.K.L. to Resources.

The authors apologise to readers for this error, which does not affect the conclusions of the article.

