# Supplementary material for: Beyond cysts – organization of epithelial networks in the murine thymus
Source: J Cell Sci. 2025 Oct 6;138(20):jcs264079. doi: 10.1242/jcs.264079 (PMC12539399; doi:10.1242/jcs.264079)
Supplement: Supplementary information [file joces-138-264079-s1.pdf]

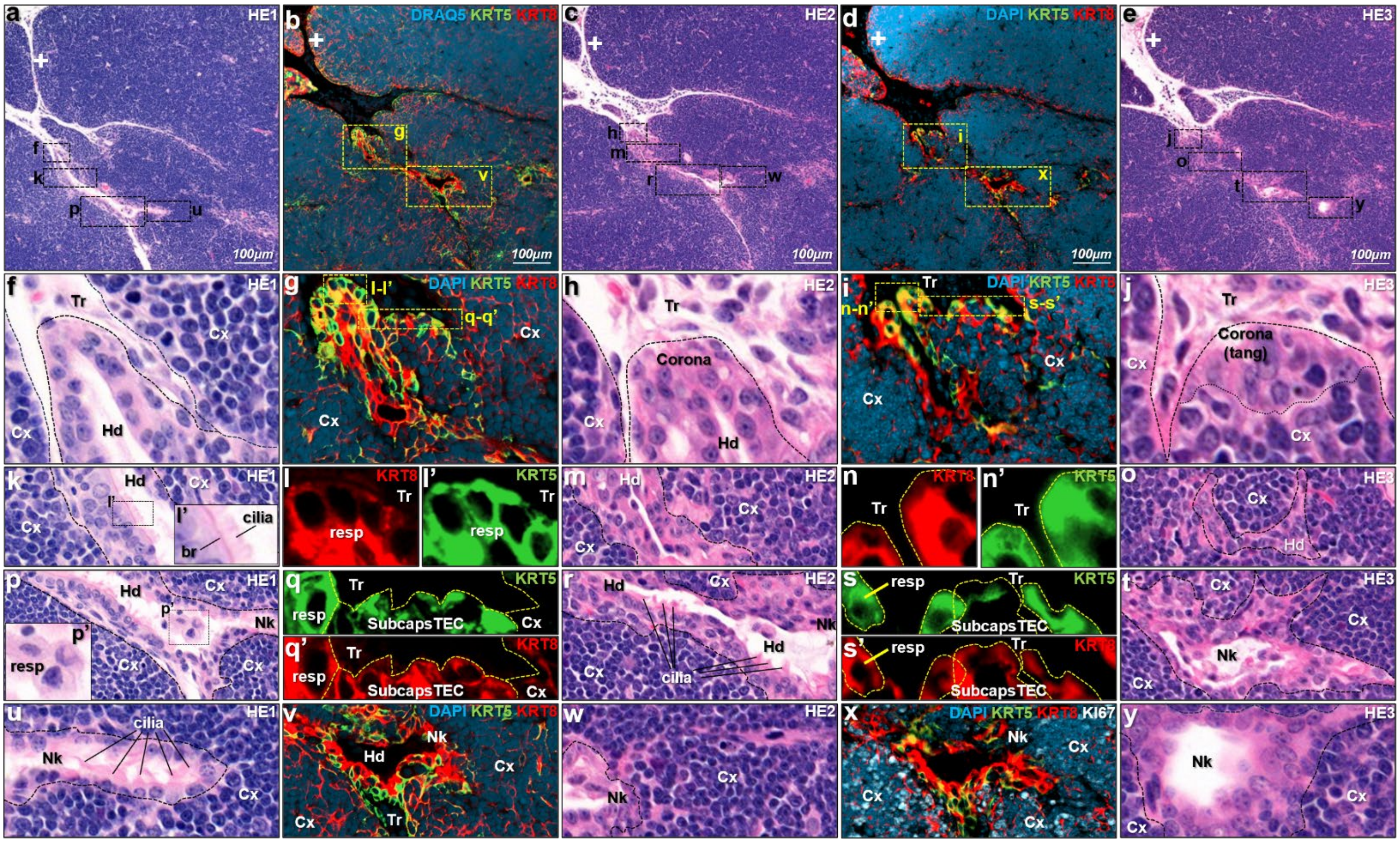

Fig. S1. Microanatomy of the Intrathymic Epithelial Network.

(a-e) Serial sections of a normal murine thymus (5-25µm apart) revealing distinct regions of the intrathymic epithelial network, including structures resembling ducts, acini, alveoli, and/or cysts, along with their microanatomical localization within the thymic lobules. Sections are stained using immunofluorescence (b, d) or H&E (a, c, e). The "+" symbol denotes corresponding microanatomical positions across sequential sections. **(f-y)** High-magnification views of selected inserts from panels (a-e), arranged in a logical sequence from the most rostral to the most caudal components of the epithelial network. These profiles highlight key features of the network.

Abbreviations: br, brush border; Cx, cortex; Hd, head; Md, medulla; Nk, neck; resp, respiratory epithelium; subcaps TEC, subcapsular thymic epithelial cells; Tr, trabeculum. Scale bars are omitted, as relative sizes can be inferred from the parental sections (a-e).

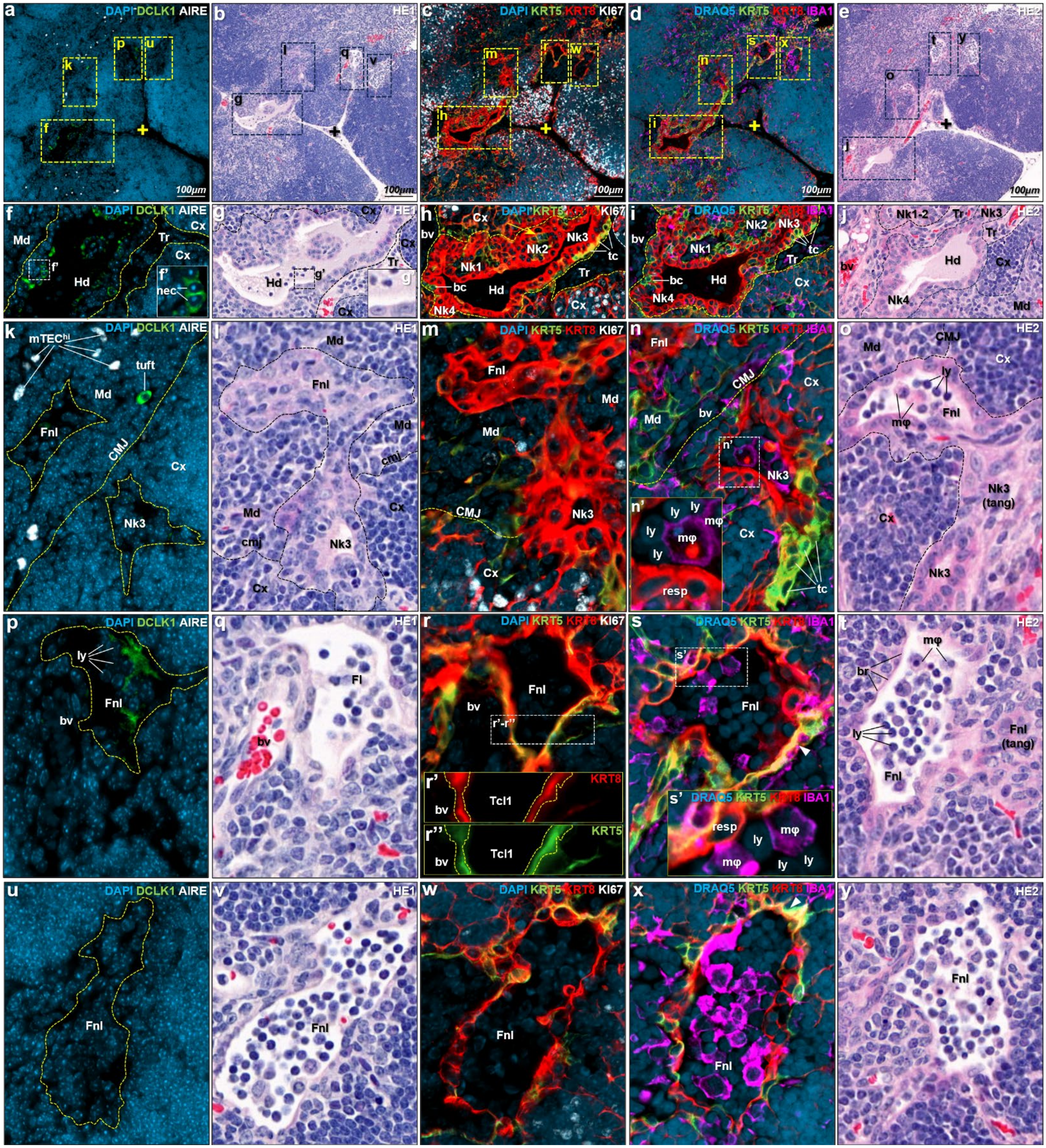

Journal of Cell Science • Supplementary information

Fig. S2. Microanatomy of the Intrathymic Epithelial Network.

(a-e) Serial sections of a normal murine thymus (5-25µm apart) revealing distinct regions of the intrathymic epithelial network, including structures resembling ducts, acini, alveoli, and/or cysts, along with their microanatomical localization within the thymic lobules. Sections are stained using immunofluorescence (a, c, d) or H&E (b, e). The "+" symbol denotes corresponding microanatomical positions across sequential sections.

(f-y) High-magnification views of selected inserts from panels (a-e), arranged in a logical sequence from the most rostral to the most caudal components of the epithelial network. These profiles highlight key features of the network.

Abbreviations: bc, basal cell; br, brush border; bv, blood vessel; Cx, cortex; CMJ, corticomedullary junction; Fnl, funnel; Hd, head; ly, lymphocyte; mφ, macrophage; Md, medulla; Nk, neck; resp, respiratory epithelium; tc, transitional cell; Tcl, tentacle; Tr, trabeculum.

Journal of Cell Science • Supplementary information

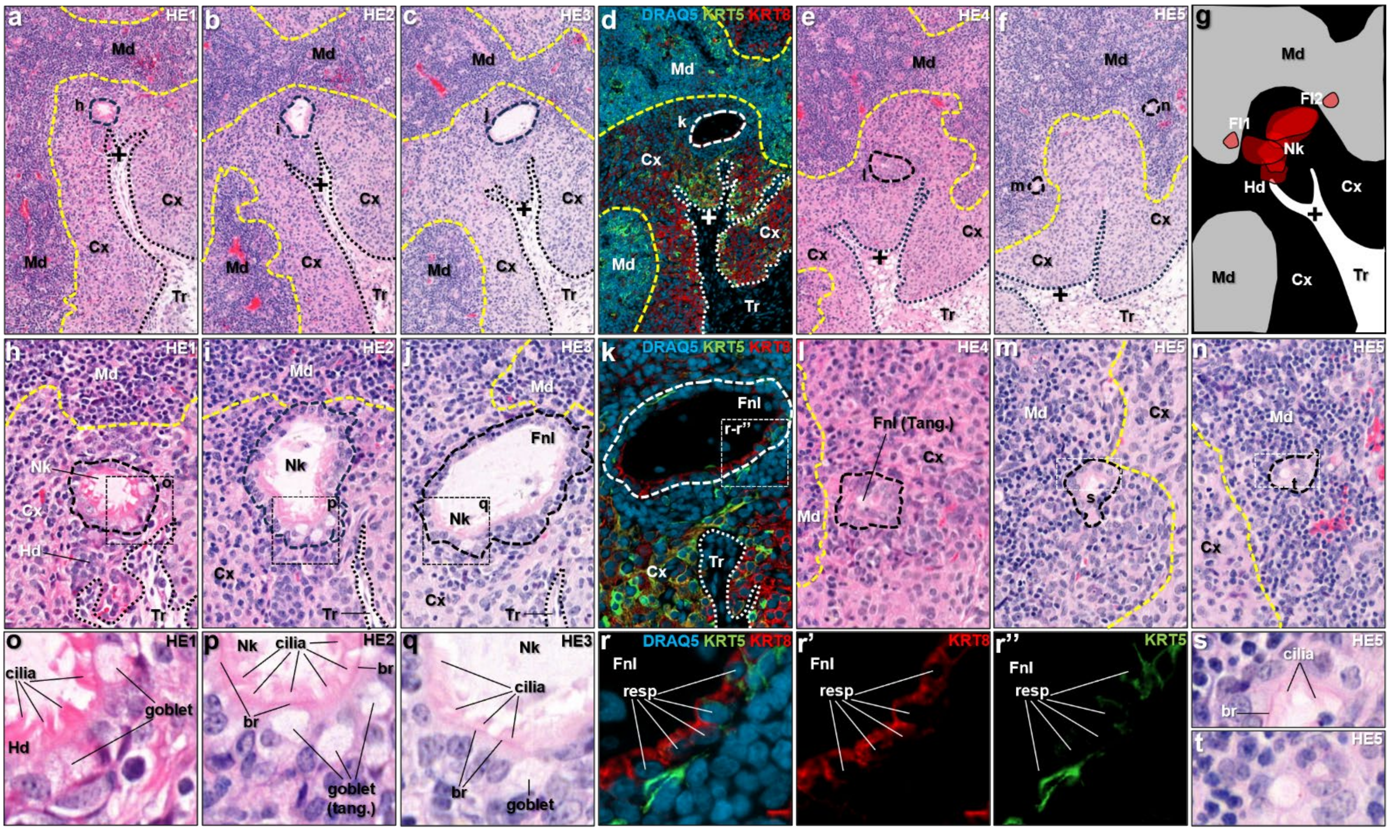

Fig. S3. Microanatomy of the Intrathymic Epithelial Network.

(a-g) Serial sections of an involuted (post-CTX day-7) murine thymus (5-25µm apart) revealing distinct regions of the intrathymic epithelial network, including structures resembling ducts, acini, alveoli, and/or cysts, along with their microanatomical localization within the thymic lobules. Sections are stained using immunofluorescence (d) or H&E (a-c & e-f). A two-dimensional pseudo-reconstruction of the network is illustrated (g), as a composite of the cystic profiles (red overlay) shown in a-f. The "+" symbol denotes corresponding microanatomical positions across sequential sections.

(f-t) High-magnification views of selected inserts from panels (a-f), arranged in a logical sequence from the most rostral to the most caudal components of the epithelial network. These profiles highlight key features of the network.

Abbreviations: br, brush border; Cx, cortex; Fnl, funnel; Hd, head; Md, medulla; Nk, neck; resp, respiratory epithelium; Tr, trabeculum.

Journal of Cell Science • Supplementary information

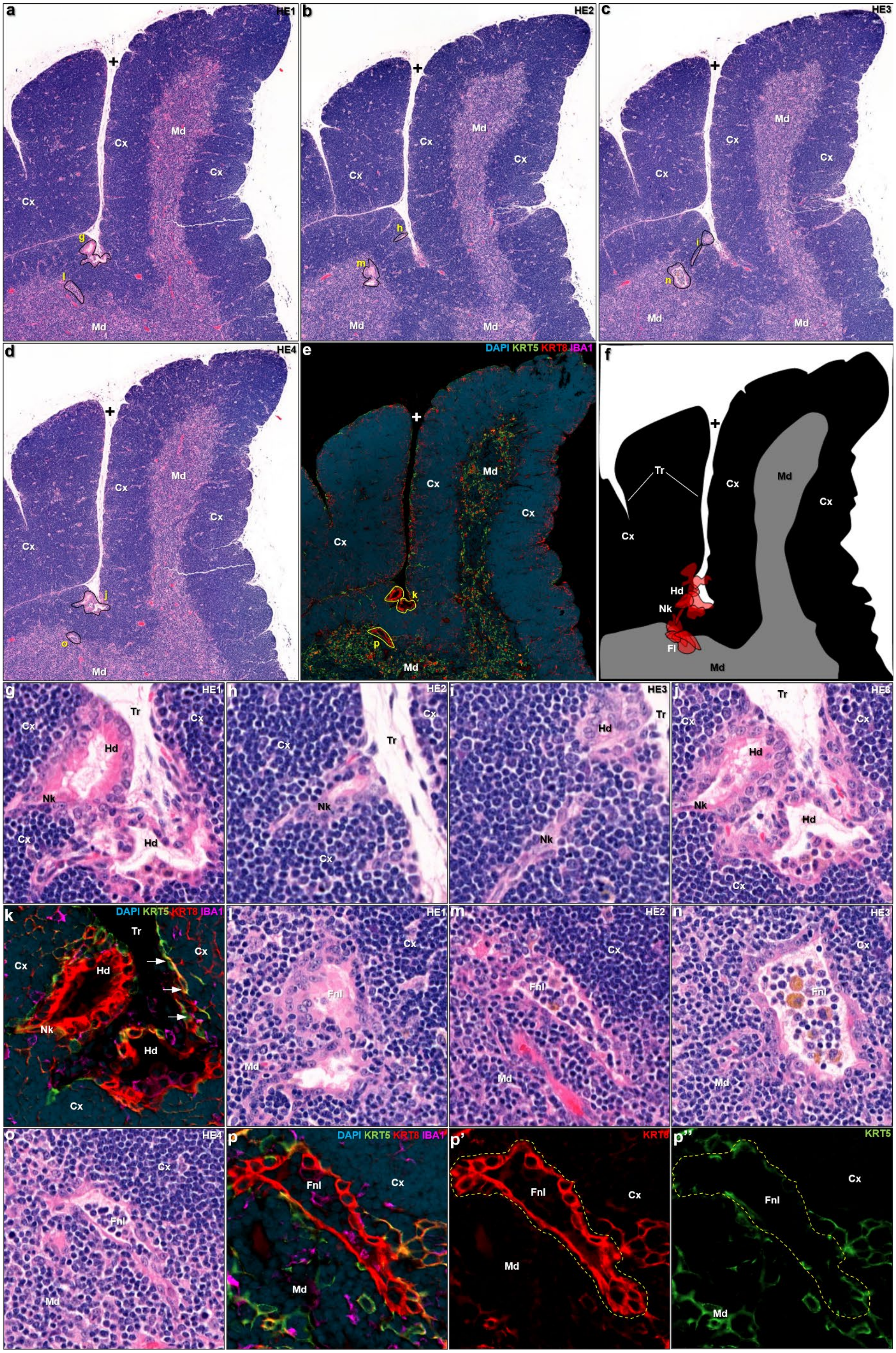

Journal of Cell Science • Supplementary information

Fig. S4. Microanatomy of the Intrathymic Epithelial Network.

(a-f) Serial sections of a normal murine thymus (5-25μm apart) revealing distinct regions of the intrathymic epithelial network, including structures resembling ducts, acini, alveoli, and/or cysts, along with their microanatomical localization within the thymic lobules. Sections are stained using immunofluorescence (e) or H&E (a-d). A two-dimensional pseudo-reconstruction of the network is illustrated (f), as a composite of the cystic profiles (red overlay) shown in a-f. The “+” symbol denotes corresponding microanatomical positions across sequential sections.

(f-p) High-magnification views of the individual cystic components from panels (a-e), arranged in a logical sequence from the most rostral to the most caudal components of the epithelial network. These profiles highlight key features of the network.

Abbreviations: Cx, cortex; Fnl, funnel; Hd, head; Md, medulla; Nk, neck; resp, Tr, trabeculum.

Journal of Cell Science • Supplementary information

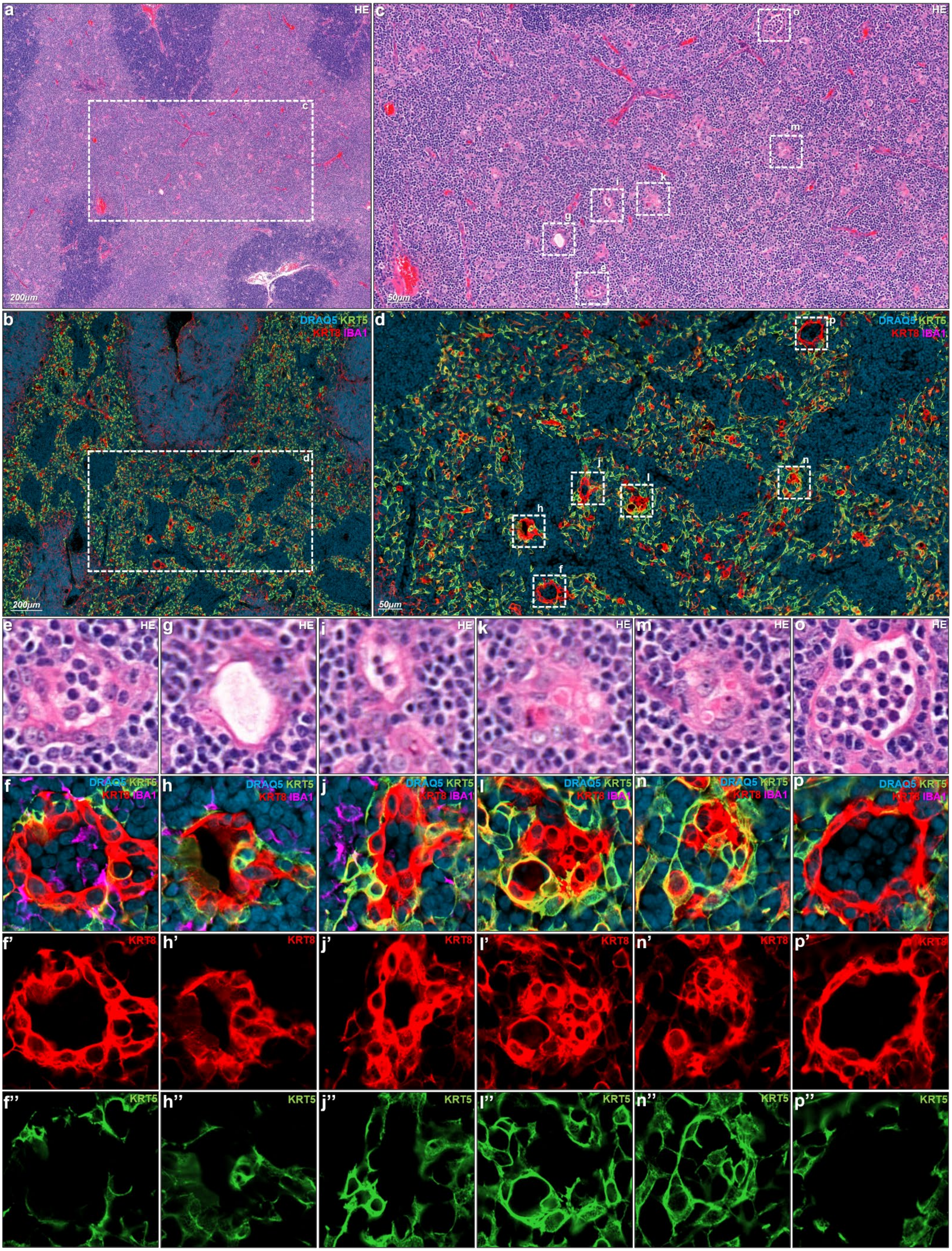
a
HE
c
200µm
b
DRAQ5 KRT5
KRT8 IBA1
d
200µm
c
HE
o
m
k
i
g
e
50µm
d
DRAQ5 KRT5
KRT8 IBA1
p
n
j
l
h
f
50µm
e
HE
g
HE
i
HE
k
HE
m
HE
o
HE
f
DRAQ5 KRT5
KRT8 IBA1
h
DRAQ5 KRT5
KRT8 IBA1
j
DRAQ5 KRT5
KRT8 IBA1
l
DRAQ5 KRT5
KRT8 IBA1
n
DRAQ5 KRT5
KRT8 IBA1
p
DRAQ5 KRT5
KRT8 IBA1
f'
KRT8
h'
KRT8
j'
KRT8
l'
KRT8
n'
KRT8
p'
KRT8
f''
KRT5
h''
KRT5
j''
KRT5
l''
KRT5
n''
KRT5
p''
KRT5

Fig. S5. Microanatomy of Epithelial Network Tentacles

(a-b) Serial sections of a normal murine thymus (spaced 5–25μm apart) showing a cluster of interconnected cystic structures in the medullary region. Sections are visualized using hematoxylin and eosin staining (H&E) (a) and immunofluorescence (b).

(c-d) Magnified views of the boxed regions in (a) and (b), respectively, highlighting six randomly distributed cystic profiles located in the deep medulla. These represent transverse sections of elongated structures referred to as “tentacles”.

(e-p) Individual representations of each of the six tentacle-like cystic profiles from panels (c) and (d), using either H&E (e, g, i, k, m, o) or immunofluorescence staining (f, h, j, l, n, p). For each profile, the hyphenated panels display the KRT8 channel, and the double-hyphenated panels show the KRT5 channel. *Abbreviations: Cx, cortex; Fnl, funnel; Hd, head; Md, medulla; Nk, neck; Tr, trabeculum. Scale bars are omitted; relative sizes can be inferred from panels (a-d).*

Journal of Cell Science • Supplementary information

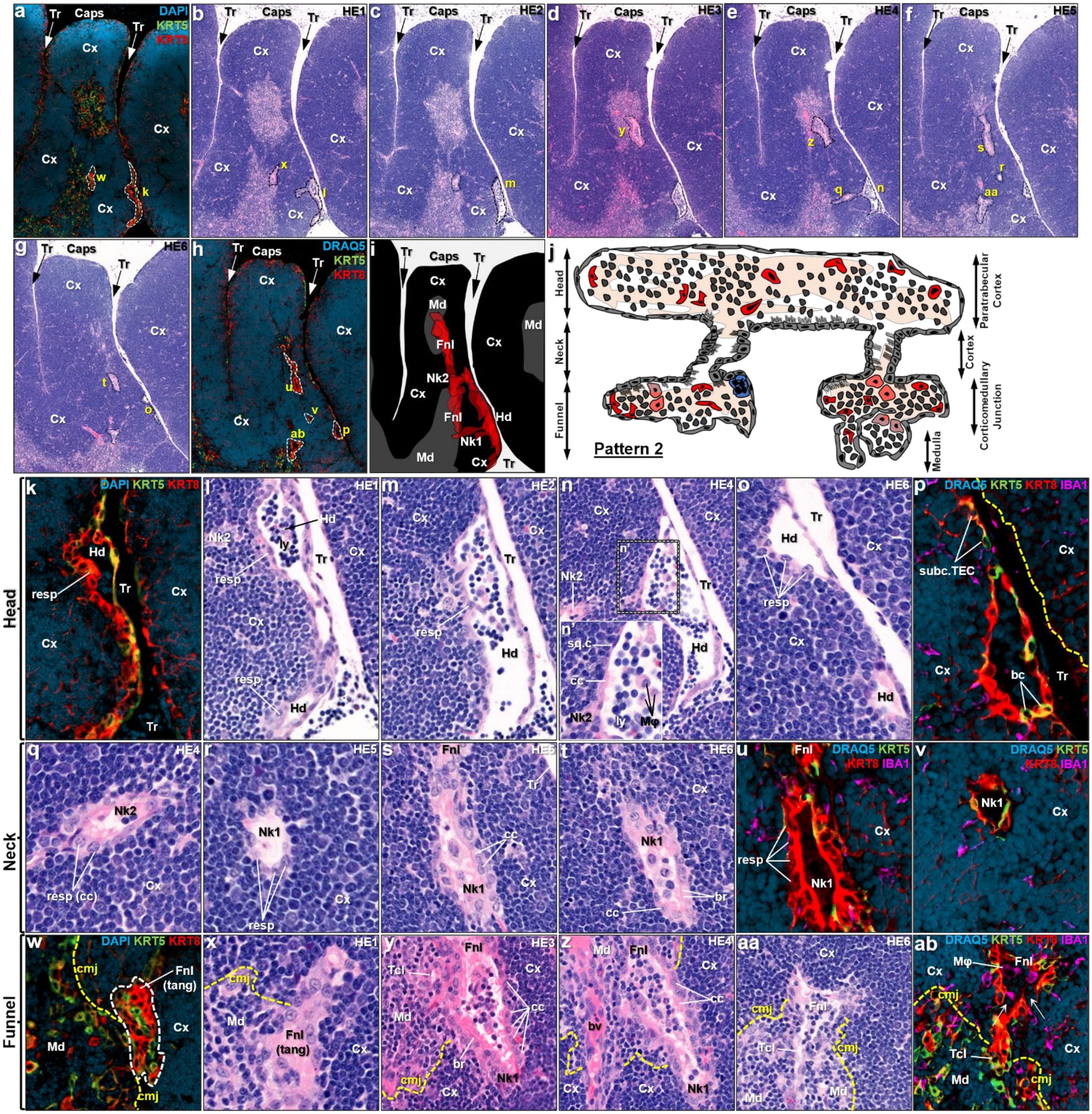

DAPI KRT5 KRT8
DRAQ5 KRT5 KRT8
DRAQ5 KRT5 KRT8 IBA1
HE1
HE2
HE3
HE4
HE5
HE6
Tr
Caps
Cx
Md
Fnl
Nk1
Nk2
Hd
Head
Neck
Funnel
Paratrabecular Cortex
Cortex
Corticomedullary Junction
Medulla
Pattern 2
resp
ly
sq.c
cc
Mφ
subc.TEC
bc
resp (cc)
br
cmj
Fnl (tang)
Tcl
bv

Fig. S6. Microanatomy of an Intrathymic Epithelial Network Variant.

(a-i) Serial sections of a normal murine thymus (5-25µm apart) revealing distinct regions of an intrathymic epithelial network representing another variant to those described in Figure 1 and Supplementary Figures 1-5, including structures resembling ducts, acini, alveoli, and/or cysts, along with their microanatomical localization within the thymic lobules. Sections are stained using immunofluorescence (a & h) or H&E (b-g). The two trabecular segments (Tr) with their arrows represent the corresponding microanatomical positions across sequential sections. A two-dimensional pseudo-reconstruction of the network is illustrated (i), as a composite of the cystic profiles (red overlay) shown in a-h.

(j) Schematic representation of the variant intrathymic epithelial network. Arrows on the left indicate anatomical portions, while arrows on the right denote predominant localization within the thymic lobule. Lining epithelial cells are shown in grey, with luminal macrophages (red), thymocytes (dark grey), and desquamated cells (blue) occupying the cavities. The sizes and shapes of the different cell types are not drawn to scale, but represent a composite model of the knowledge obtained through the manuscript.

(k-p) Magnified views of the cystic profiles in the Head portion, as visualized in different planes using immunofluorescence (k, p) or H&E (l-o).

(q–v) Magnified views of the cystic profiles in the Neck portion, as visualized in different planes using immunofluorescence (u, v) or H&E (q-t).

(q–ab) Magnified views of the cystic profiles in the Funnel portion, as visualized in different planes using immunofluorescence (q, ab) or H&E (x-aa).

Abbreviations: br, brush border; bv, blood vessel; cc, cuboidal cell; cmj, corticomedullary junction; Cx, cortex; Fnl, funnel; Hd, head; ly, lymphocyte; Md, medulla; Nk, neck; resp, respiratory epithelium; Subc. TEC, subcapsular TEC; Tcl, tentacle, Tr, trabeculum. Scale bars are omitted; relative sizes can be inferred from panels (a-d).

Journal of Cell Science • Supplementary information

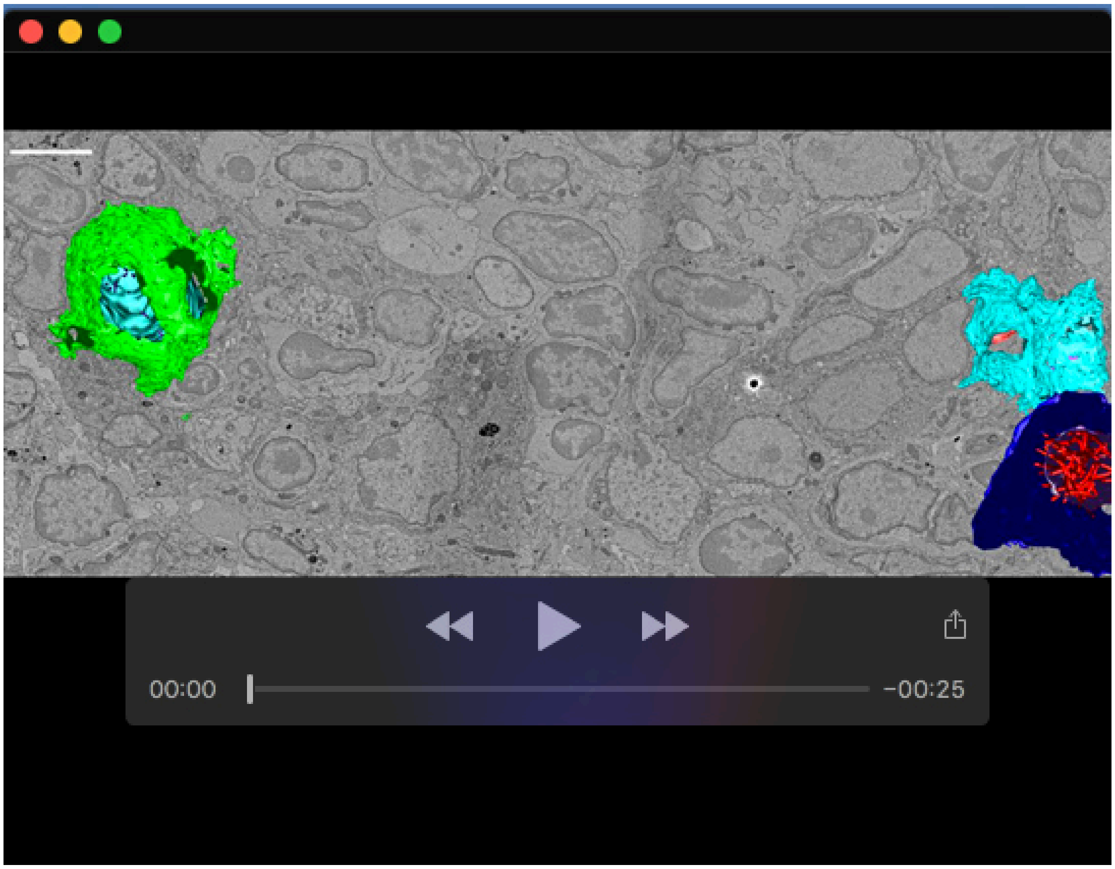

Movie 1. Three-dimensional reconstruction of three solitary ciliated cells in the murine medulla, using OTO-SEM 3D Array Tomography, at post-chemotherapy day-14. Cell membranes are shown in green, light blue, and dark blue; lumen walls are shown in cyan, red, and pink; cilia are shown in purple, yellow, and red; nuclei are shown in blue, and purple. Scale bar is 5um.
